# Supplementary material for: The Impact of Person-Centered Care Indicators on Care Strain Among Care Aides in Long-Term Care Homes in New Brunswick: A Cross-Sectional Study
Source: Nurs Rep. 2025 Apr 26;15(5):140. doi: 10.3390/nursrep15050140 (PMC12114448; doi:10.3390/nursrep15050140)
Supplement: Supplementary file 1 [file nursrep-15-00140-s001.zip › nursrep-3481657-supplementary.pdf]

nursing reports

MDPI

Supplementary Material S1: Linear models of the association between PCC indicators and strain in dementia care

PCC indicator	T-value	SE	Cohen's f^2	P-value	Power
1. Preferences of residents are recorded systematically and in a structured way					
Present	Reference				
Absent	-0.361	1.631	0.006	0.721	0.639
Unsure	0.215	1.324	0.002	0.832	0.584
2. There are policies/procedures in place on how to deal with refusals of care.					
Present	Reference				
Absent	-0.262	1.619	0.003	0.769	0.602
Unsure	1.445	1.442	0.099	0.163	0.918
3. Mandatory education and training on person-centered care is required for all staff.					
Present	Reference				
Absent	3.968	1.196	0.750	**<0.001**	0.999
Unsure	0.944	1.533	0.042	0.356	0.822
4. A process is in place to provide guidance and support for staff dealing with ethically challenging resident care issues.					
Present	Reference				
Absent	3.828	1.139	0.698	**<0.001**	0.999
Unsure	0.718	1.209	0.025	0.481	0.759

Supplementary Material S2: Linear models of the association between PCC indicators and care aide daily emotions

PCC indicator	T-value	SE	Cohen's f^2	P-value	Power
1. Preferences of residents are recorded systematically and in a structured way					
Present	Reference				
Absent	0.486	3.368	0.010	0.631	0.684
Unsure	-0.321	2.718	0.004	0.751	0.624
2. There are policies/procedures in place on how to deal with refusals of care.					
Present	Reference				
Absent	-0.346	3.427	0.005	0.732	0.634
Unsure	-1.372	3.048	0.078	0.183	0.909
3. Mandatory education and training on person-centered care is required for all staff.					
Present	Reference				
Absent	-3.368	2.666	0.473	**0.003**	0.999
Unsure	-1.598	3.428	0.106	0.123	0.938
4. A process is in place to provide guidance and support for staff dealing with ethically challenging resident care issues.					
Present	Reference				
Absent	-4.002	2.364	0.667	**<0.001**	0.999
Unsure	-1.474	2.533	0.091	0.153	0.923
